# Tolvaptan and Autosomal Dominant Polycystic Kidney Disease Progression in Individuals Aged 18-35 Years: A Pooled Database Analysis

**DOI:** 10.1016/j.xkme.2024.100935

**Published:** 2024-11-14

**Authors:** Fouad T. Chebib, Neera Dahl, Xiaolei Zhou, Diana Garbinsky, Jinyi Wang, Sasikiran Nunna, Dorothee Oberdhan, Ancilla W. Fernandes

**Affiliations:** 1 Mayo Clinic Division of Nephrology and Hypertension, Jacksonville, FL; 2 Mayo Clinic Division of Nephrology and Hypertension, Rochester, MN; 3 RTI Health Solutions, Research Triangle Park, NC; 4 Otsuka Pharmaceutical Development & Commercialization, Inc, Rockville, MD

**Keywords:** Autosomal dominant polycystic kidney disease, estimated glomerular filtration rate, kidney failure, pooled database analysis, tolvaptan, young adults

## Abstract

**Rational & Objective:**

Data are limited regarding the long-term efficacy of tolvaptan in adults aged 18-35 years with autosomal dominant polycystic kidney disease (ADPKD) at increased risk of rapid progression. We assessed the effects of tolvaptan within a larger population of younger adults and over longer follow-up than individual clinical trials could provide.

**Study Design:**

Pooled database study.

**Setting & Study Populations:**

A consolidated clinical study database with ADPKD patients aged 18-35 years.

**Selection Criteria for Studies:**

Studies that enrolled patients who received either tolvaptan or standard-of-care treatment not including tolvaptan.

**Data Extraction:**

Annual rate of change in estimated glomerular filtration rate (eGFR) and time to kidney failure.

**Analytical Approach:**

For individuals participating in multiple studies, their data were longitudinally linked to extend the follow-up period. We matched tolvaptan-treated patients with controls based on age, sex, chronic kidney disease stage, eGFR, and, where possible, Mayo Imaging Classification. We compared eGFR decline between groups using mixed-effects modeling.

**Results:**

The matched analysis set encompassed 204 tolvaptan-treated individuals and 204 controls. Median follow-up was 4.6 years for the tolvaptan group and 1.7 years for controls. In the mixed-effects model, the eGFR decline rate (in mL/min/1.73 m^2^/year) was –2.58 for the tolvaptan cohort and -4.28 for controls. This indicates reduction in the eGFR decline rate by 1.69 mL/min/1.73 m^2^/year (95% confidence interval: 0.87-2.52; *P* < 0.001) with tolvaptan, a 40% improvement. Extrapolating eGFR over 35 years, tolvaptan could delay kidney failure onset by approximately 11 years.

**Limitations:**

Median follow-up was shorter in the control cohort than the tolvaptan cohort. The projection of time to kidney failure assumed a linear model of eGFR decline.

**Conclusions:**

This analysis offers insights into the anticipated treatment benefits of tolvaptan for young adults with ADPKD. These findings are crucial for weighing treatment benefits against any associated risks.

## Plain-Language Summary

Tolvaptan is the only approved treatment for delaying kidney function decline in patients with autosomal dominant polycystic kidney disease (ADPKD) at high risk of rapid progression. Clinical trials have included few patients aged 18-35 years, a group potentially benefiting significantly from early tolvaptan initiation. We pooled clinical study data, matching tolvaptan-treated patients with untreated controls by baseline characteristics. The results showed a statistically significant reduction in kidney function decline with tolvaptan. Extrapolating this 5-year data to a 35-year disease trajectory suggests an 11-year delay in the onset of kidney failure. This analysis underscores the potential long-term benefits of early treatment with tolvaptan in younger ADPKD patients.

In the pivotal clinical trials Tolvaptan Efficacy and Safety in Management of Autosomal Dominant Polycystic Kidney Disease and Its Outcomes 3:4 (TEMPO 3:4; NCT00428948) and Replicating Evidence of Preserved Renal Function: an Investigation of Tolvaptan Safety and Efficacy in ADPKD (REPRISE; NCT02160145), the vasopressin V2 receptor antagonist tolvaptan significantly slowed kidney function decline versus placebo in patients with autosomal dominant polycystic kidney disease (ADPKD) who were at elevated risk of rapid disease progression.^1^^,^^2^ The trials recruited populations across a range of ages and disease progression stages. The 3-year TEMPO 3:4 trial enrolled participants aged 18-50 years with total kidney volume (TKV) ≥750 mL and preserved kidney function (creatinine clearance ≥60 mL/min).^1^ The 1-year REPRISE trial enrolled participants who were, on average, older and with more advanced kidney function decline. Eligibility criteria for REPRISE included ages 18-55 years with an estimated glomerular filtration rate (eGFR) of 25-65 mL/min/1.73 m^2^ or ages 56–65 years with an eGFR of 25-44 mL/min/1.73 m^2^ and evidence of rapid eGFR decline.^2^

Patients considering tolvaptan initiation are counseled that treatment typically requires long-term adherence, and to understand the risks and benefits. The adverse effects associated with tolvaptan are well known, and include aquaresis and increased risk for potentially serious liver enzyme elevations.^3^ Such considerations need to be weighed against evidence that earlier treatment initiation is associated with a greater cumulative impact on disease progression. Participants who received placebo in TEMPO 3:4 and initiated tolvaptan at the start of the 2-year TEMPO 4:4 (NCT01214421) open-label extension experienced a decreased rate of kidney function decline during the extension. However, the kidney function benefit was lower compared with participants who received tolvaptan for 3 years in TEMPO 3:4 and for another 2 years in TEMPO 4:4.^4^ The potential benefits of earlier initiation are especially relevant to younger patients, who could slow disease progression over a longer period and thereby potentially experience greater cumulative improvement in ADPKD outcomes than older patients.^5^ In a single-center experience with long-term follow-up (up to 11.2 years, average 4.6 years), the beneficial effect of tolvaptan was shown to be sustained and cumulative as compared with matched controls.^6^ Thus, it might be prudent to start the treatment as early as possible for maximal beneficial effect.

The REPRISE trial, primarily enrolling an older population (mean age: 47 years), offered limited insights into tolvaptan's efficacy in young adults aged 18-35 years.^2^ Subgroup analyses of the TEMPO 3:4 data indicated a slightly greater benefit in patients aged 35 years or older compared with younger individuals.^1^ Subgroup analyses of randomized, controlled trials can introduce imbalances in patient characteristics, highlighting the need for alternative analytical methods to accurately assess treatment efficacy in specific subpopulations.^7^ To better understand the long-term effects of tolvaptan on kidney function decline in young adults, we analyzed data from a pooled database of multiple clinical studies in ADPKD, leveraging techniques such as long-term data linkage, patient matching, and mixed-effects modeling to assess treatment impact on kidney function over time.

## Methods

### Data Source and Patient Eligibility Criteria

This study used a pooled database of clinical trials of tolvaptan and ADPKD clinical studies in which patients did not receive tolvaptan. The studies included in the database have been described by Zhou et al.^8^ The database includes 2,928 patients with ADPKD who were treated with tolvaptan and 4,189 who received standard-of-care treatment for ADPKD not involving tolvaptan. Data on individual patients who participated in one study and entered into a subsequent study were linked longitudinally to enable longer duration of follow-up.

**Figure 1:**
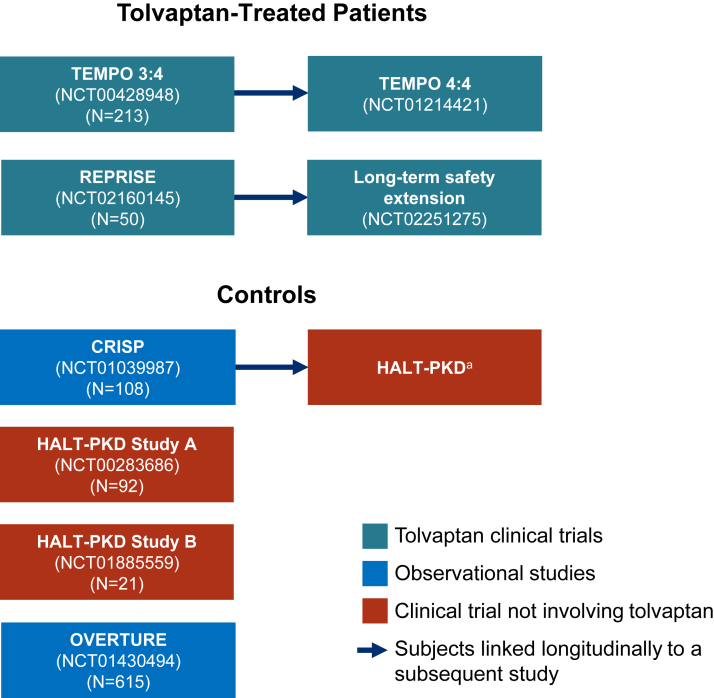
Source studies for the pooled dataset of eligible patients aged 18-35 years. The tolvaptan cohort included patients randomized to the tolvaptan arm and excluded patients enrolled in Japan in TEMPO 3:4. The non-tolvaptan cohort excluded patients who used tolvaptan, were linked in an early study, were randomized to the low blood pressure control arm of HALT-PKD Study A, or were enrolled in Japan in OVERTURE. ^a^Data were linked for a small proportion of CRISP participants who later participated in HALT-PKD.

Patients with a baseline age of 18-35 years and who had a baseline and ≥1 postbaseline eGFR assessment were eligible for inclusion in the present analysis and comprised the full analysis set. The young adults treated with tolvaptan and included in the analysis were selected from the TEMPO 3:4 and REPRISE trials, along with their respective extension studies (Fig 1). The non-tolvaptan-treated comparator group (controls) participated in the Consortium for Radiologic Imaging Studies of Polycystic Kidney Disease (CRISP I and II; NCT01039987), HALT Progression of Polycystic Kidney Disease (HALT-PKD) Study A (NCT00283686) or Study B (NCT01885559), or the OVERTURE study (NCT01430494).^9^, ^10^, ^11^, ^12^ CRISP is a noninterventional study, the HALT-PKD studies evaluated various antihypertensive regimens, and OVERTURE was an observational study of ADPKD patients receiving standard-of-care treatment before tolvaptan availability for ADPKD.

### Matching Procedure

To reduce confounding in the comparison of tolvaptan-treated patients with controls not on tolvaptan, matching was performed among participants in the full analysis set. The matching criteria included baseline characteristics: age (within a ±2-year range), sex (either female or male), chronic kidney disease (CKD) stage (G1 to G5), eGFR (within ±5 mL/min/1.73 m^2^), and, where available, Mayo Imaging Classification (1A to 1E; based on age and height-adjusted TKV).^13^ Tolvaptan-treated patients and controls were matched 1:1 using the %GMATCH SAS macro developed by the Mayo Clinic, which uses a greedy matching algorithm.^14^

Table 1 displays the matching procedure, which was performed in steps because of different eGFR requirements and availability of TKV in the source studies (TKV was not available in REPRISE or HALT-PKD Study B). For the non-tolvaptan-treated controls, priority was given to those from CRISP and HALT-PKD, which had longer follow-up than OVERTURE.

**Table 1:** Matching Procedure for the Matched Analysis Set

Step	Matching Method
Step 1	Match patients from REPRISE to HALT-PKD Study B on CKD stage (G1, G2, G3, G4, G5), sex, age (±2 years), and baseline enzymatic eGFR (±5 mL/min/1.73 m^2^)
Step 2	Match patients from TEMPO 3:4 to CRISP and HALT-PKD Study A on CKD stage, sex, age, baseline enzymatic eGFR, Mayo Imaging Class (1A, 1B, 1C, 1D, 1E), and duration of follow-up (±9 months; patients with a follow-up longer than 5 years were truncated at 5 years for matching)
Step 3	Match the remaining patients from TEMPO 3:4 to OVERTURE on CKD stage, sex, age, baseline enzymatic eGFR, and Mayo Imaging Class
Step 4	Match the remaining patients from REPRISE to the remaining patients from CRISP, HALT-PKD Study A, and OVERTURE on CKD stage, sex, age, and baseline enzymatic eGFR

CKD, chronic kidney disease; eGFR, estimated glomerular filtration rate.

Note: Mayo Imaging Class was not available in REPRISE and HALT-PKD Study B.

### Outcomes

The primary outcome of interest in this study was to investigate the impact of tolvaptan treatment, in comparison to non-tolvaptan standard of care alone, on the annual rate of change in eGFR. In the tolvaptan-treated group, we excluded eGFR assessments conducted <7 days after tolvaptan initiation, during tolvaptan treatment gaps, or after discontinuation of tolvaptan treatment. This exclusion aimed to eliminate the acute hemodynamic effects of tolvaptan, characterized by a rapid decrease in eGFR following treatment initiation, which typically reverse after discontinuation.^15^^,^^16^ Furthermore, for patients initially enrolled in CRISP who subsequently participated in the HALT-PKD low blood pressure control arm, eGFR assessments conducted during their involvement in HALT-PKD were also excluded.

Delay in time to kidney failure (ie, eGFR <15 mL/min/1.73 m^2^) with tolvaptan was also estimated for the purpose of illustration, as described in the statistical analyses section below.

### Statistical Analyses

Descriptive statistics are presented for baseline patient characteristics, and duration of tolvaptan exposure and follow-up in the matched analysis set. The annual rate of change in eGFR in the matched sets was estimated using a mixed model that included time (continuous), treatment, baseline eGFR, and time-by-treatment interaction as fixed effects, and patient-specific intercepts and slopes (for time) as random effects with an unstructured variance-covariance matrix. Change from “baseline” eGFR was estimated based on the theoretical baseline value estimated from the mixed model. To reduce potential bias caused by informative missingness (fewer patients had data after 5.5 years), eGFR measurements after 5.5 years were excluded for the mixed model analysis. Because of a difference in duration of follow-up between the matched tolvaptan and control cohorts, mostly because of the short duration of follow-up of the OVERTURE study, a sensitivity analysis was conducted using the same mixed-model methodology but that included matched controls only from the CRISP and HALT-PKD studies and excluded those from OVERTURE.

To illustrate the delay in progression to kidney failure among young adults initiating tolvaptan, we conducted extrapolations of predicted eGFR values for up to 35 years. These extrapolations assumed a similar relationship to that observed during the initial 5.5 years, with a baseline eGFR of 93 mL/min/1.73 m^2^ (which corresponds to the sample mean of the matched analysis set). The estimation of 95% prediction intervals was accomplished using the empirical best linear unbiased predictor.

All analyses were conducted using observed data without imputation for missing values.

### Ethical Conduct

This study did not enroll human subjects and was exempted from Institutional Review Board approval. All the data collected were provided in deidentified and/or anonymized form and, as such, informed consent was not required.

## Results

### Analysis Population

In our study, a total of 1,099 patients were included, with 263 receiving tolvaptan treatment and 836 serving as controls. These patients were aged 18-35 years, and had a baseline and at least one postbaseline eGFR assessment. Among these eligible patients, 408 individuals (204 tolvaptan treated and 204 controls) were paired through our matching procedure. The specific numbers matched at each step are provided in Supplementary Table S1. Additionally, the breakdown by source study is detailed in that table, indicating that in the tolvaptan cohort, there were 180 patients from TEMPO 3:4 and 24 from REPRISE. For the control (standard-of-care) cohort, there were 144 patients from OVERTURE, 23 from CRISP, 30 from HALT-PKD Study A, and 7 from HALT-PKD Study B.

Baseline characteristics used for the matching were balanced as expected between the two cohorts and other characteristics were generally well balanced (Table 2).^17^ The mean age in both cohorts was 30 years and the average (mean) age at ADPKD diagnosis was 21 years (median 21 years). The tolvaptan cohort had more patients who identified as White (93.1%) and fewer who identified as Hispanic (3.9%) relative to the control cohort (76.5% White, 15.2% Hispanic). The mean eGFR was 93 mL/min/1.73 m^2^ in both treatment groups. The distribution of CKD stages was as follows: G1, 62.3%; G2, 23.0%; G3a, 8.3%; G3b, 4.9%; G4, 1.5%; G5, 0.0%. Mayo Imaging Class was available for 180 (88.2%) of the matched patients in the tolvaptan cohort, with the class distribution indicating a population at high risk of rapid progression: 1A, 0.0%; 1B, 0.0%; 1C, 26.1%; 1D, 36.7%; 1E, 37.2%.

**Table 2:** Patient Baseline Characteristics of the Matched Set

Characteristic	Tolvaptan (n = 204)	Non-Tolvaptan-Treated Controls (n = 204)	Standardized Mean Difference^d^
Age (y), mean (SD)	30.2 (4.1)	30.1 (4.1)	0.01
Range	18.7-35.0	18.7-35.0	
Sex, n (%)			
Female	92 (45.1)	92 (45.1)	0.00
Male	112 (54.9)	112 (54.9)	0.00
Race, n (%)			
Asian	1 (0.5)	7 (3.4)	–0.21
Black	5 (2.5)	3 (1.5)	0.07
Hispanic	8 (3.9)	31 (15.2)	–0.39
White	190 (93.1)	156 (76.5)	0.48
Combined^a^	0 (0.0)	7 (3.4)	–0.27
Body mass index in kg/m^2^, mean (SD)	26.7 (7.3)	26.6 (5.6)	0.03
Age at ADPKD diagnosis in years, mean (SD)	21.1 (7.2)	21.1 (7.9)	0.01
Median (IQR)	21.5 (16.0-26.0)	21.3 (17.0-26.6)	
CKD stage,^b^ n (%)			
G1	127 (62.3)	127 (62.3)	0.00
G2	47 (23.0)	47 (23.0)	0.00
G3a	17 (8.3)	17 (8.3)	0.00
G3b	10 (4.9)	10 (4.9)	0.00
G4	3 (1.5)	3 (1.5)	0.00
G5	0 (0.0)	0 (0.0)	
eGFR in mL/min/1.73 m^2^, mean (SD)	92.7 (25.1)	92.7 (25.3)	-0.00
Systolic blood pressure in mm Hg, mean (SD)	128.2 (13.6)	127.9 (12.8)	0.02
Diastolic blood pressure in mm Hg, mean (SD)	81.6 (9.2)	81.1 (9.5)	0.05
History of nephrolithiasis, n (%)	36 (17.6)	14 (6.9)	0.33
History of hematuria, n (%)	68 (33.3)	32 (15.7)	0.42
History of urinary tract infection, n (%)	62 (30.4)	15 (7.4)	0.62
TKV in mL, n^c^	180	180	
Mean (SD)	1484.8 (884.3)	1421.2 (688.1)	0.08
Median (IQR)	1222.0 (944.7-1665.8)	1250.8 (929.7-1657.1)	
Height-adjusted TKV in mL/m, n (%)^c^	180	180	
<400	0 (0.0)	6 (3.3)	–0.26
400 to <600	66 (36.7)	55 (30.6)	0.13
≥600	114 (63.3)	119 (66.1)	–0.06
Mayo Imaging Class, n (%)^c^	180	180	
1A	0 (0.0)	0 (0.0)	
1B	0 (0.0)	0 (0.0)	
1C	47 (26.1)	47 (26.1)	0.00
1D	66 (36.7)	66 (36.7)	0.00
1E	67 (37.2)	67 (37.2)	0.00
Duration of follow-up in years, median (min, max)	4.6 (0.0, 5.5)	1.7 (0.3, 5.5)	e

ADPKD, autosomal dominant polycystic kidney disease; CKD, chronic kidney disease; eGFR, estimated glomerular filtration rate; IQR, interquartile range; max, maximum; min, minimum; SD, standard deviation; TKV, total kidney volume.

a Includes American Indian or Alaska Native, Native Hawaiian or other Pacific Islander, and a race or ethnicity not listed.

b Stage G1, ≥90 mL/min/1.73 m^2^; stage G2, 60 to <90 mL/min/1.73 m^2^; stage G3a, 45 to <60 mL/min/1.73 m^2^; stage G3b, 30 to <45 mL/min/1.73 m^2^; stage G4, 15 to <30 mL/min/1.73 m^2^; stage G5, <15 mL/min/1.73 m^2^.

c The control cohort was restricted to patients whose matched tolvaptan counterparts had nonmissing data.

d Values >0.2 were considered to be indicative of between-group differences. On the interpretation of standardized mean .difference, see Austin PC.^17^

e Standardized mean difference between the treatment cohorts was not calculated, as duration of follow-up is not a baseline characteristic.

### Exposure and Follow-up in the Matched Cohorts

Within the tolvaptan cohort, the average (mean) duration of treatment was 4.4 years (standard deviation 2.7) with a mean compliance rate of 94.7% (calculated as the number of on-treatment days divided by treatment duration, multiplied by 100%). For the tolvaptan cohort, the median duration of eGFR follow-up was 4.6 years (range 0.0-5.5). In contrast, the control cohort had a median duration of eGFR follow-up of 1.7 years (range 0.3-5.5). The tolvaptan cohort had a mean duration of eGFR follow-up of 3.5 years with a standard deviation of 2.0 years, whereas the control cohort had a mean duration of 2.3 years with a standard deviation of 1.4 years.

### Estimated Tolvaptan Effects on Kidney Function Decline and Time to Kidney Failure

**Figure 2:**
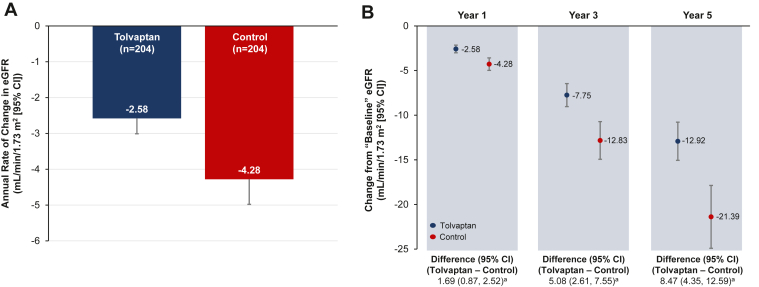
Estimates from the mixed model for **(A)** annual rate of eGFR change and **(B)** cumulative eGFR change at years 1, 3, and 5 from “baseline” in the tolvaptan and control cohorts. For panel B, change from “baseline” eGFR was calculated based on the theoretical baseline value estimated from the mixed model. ^a^*P* < 0.001. CI, confidence interval; eGFR, estimated glomerular filtration rate.

The mixed model estimated the annual rate of decline in eGFR in mL/min/1.73 m^2^ (with a 95% confidence interval [CI]) to be –2.58 (–3.01 to –2.15) for the tolvaptan cohort and –4.28 (–4.98, –3.57) for the matched control cohort (Fig 2A). Notably, tolvaptan significantly reduced the annual rate of eGFR decline by 1.69 mL/min/1.73 m^2^ (95% CI 0.87-2.52; *P* < 0.001; Fig 2B), corresponding to a 40% decrease. The annual difference translated to a 5-year cumulative slowing in eGFR decline of 8.47 mL/min/1.73 m^2^ (95% CI 4.35-12.59) by tolvaptan.

**Figure 3:**
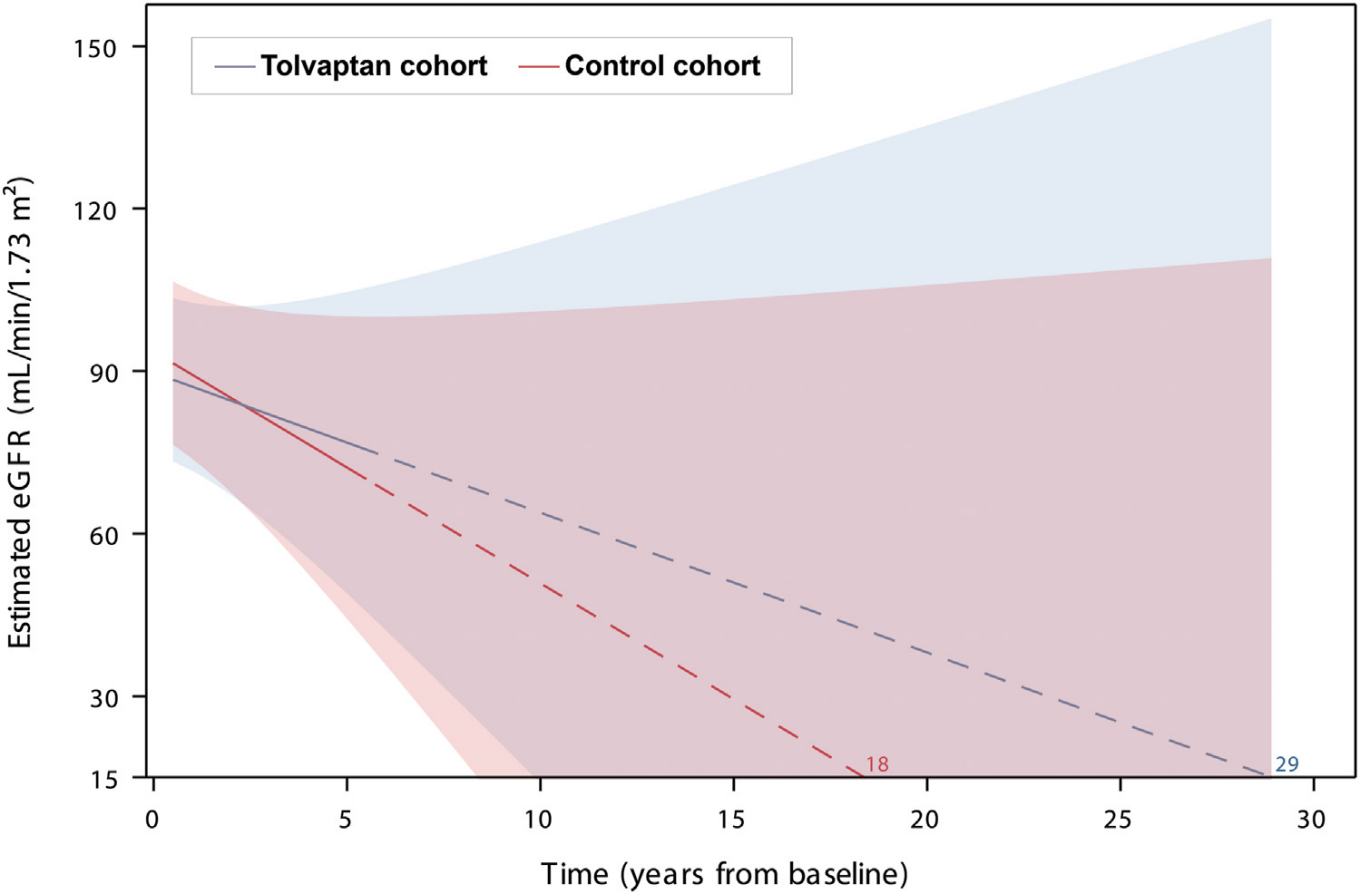
Extrapolations of predicted eGFR values and time to kidney failure (eGFR <15 mL/min/1.73 m^2^) from the mixed model. Extrapolations were based on the mixed model including treatment, time (continuous), treatment-by-time interaction, and baseline eGFR for a patient with baseline eGFR at 93 mL/min/1.73 m^2^. Solid lines represent observed values to 5.5 years and dotted lines represent the extrapolated values. The shaded 95% prediction intervals were based on the empirical best linear unbiased predictor, which accounted for additional variability compared with the estimation on observed data. Extrapolations are presented for illustration purposes, and assume maintenance of treatment effect and the linear relationship over time, which may not be true for all patients (eg, patients with different Mayo Imaging Classes). These projections should be interpreted with caution. eGFR, estimated glomerular filtration rate.

Extrapolating eGFR data from the mixed model for patients with a baseline eGFR of 93 mL/min/1.73 m^2^ and assuming a relationship consistent with that observed in the first 5.5 years, patients receiving tolvaptan were predicted to reach kidney failure onset (eGFR <15 mL/min/1.73 m^2^) approximately 11 years later than patients receiving standard of care alone (Fig 3).

### Sensitivity Analysis Excluding Participants in OVERTURE

Given the difference in median eGFR duration of follow-up between the tolvaptan cohort (4.6 years) and the control cohort (1.7 years) within the matched population, mostly because of the short duration of follow-up of the OVERTURE study (designed to measure the study primary endpoint between 11 and 19 months), a sensitivity analysis was performed that included controls only from the CRISP and HALT-PKD studies, and excluded those from OVERTURE. The sensitivity analysis was otherwise conducted using the same procedures as the original matched analysis. A total of 60 tolvaptan-treated patients were matched to 60 controls and both cohorts were similarly well balanced for baseline characteristics as in the original matched analysis (Supplementary Table S2). Median duration of eGFR follow-up was 5.3 years (range 0.1-5.5) for the tolvaptan cohort and 5.0 years (range 0.3-5.5) for the control cohort. As in the main analysis of matched cohorts, the sensitivity analysis indicated a significant difference in eGFR decline for the tolvaptan cohort versus the control cohort, both in terms of annual rate of decline (slowing of decline by 1.15 mL/min/1.73 m^2^ [95% CI 0.04-2.25; *P* = 0.04] with tolvaptan) and the cumulative difference (5.73 mL/min/1.73 m^2^ [95% CI 0.20-11.26] less decline with tolvaptan over 5 years) (Supplementary Fig S1).

## Discussion

The results of our analysis strongly support a significant delay in kidney function decline and a prolongation of time to kidney failure in young adults (ages 18-35 years) at elevated risk of rapid ADPKD progression when treated with tolvaptan. The statistical model showed that the annual rate of eGFR decline was 40% slower in the tolvaptan cohort (2.58 mL/min/1.73 m^2^) compared with the control cohort (4.28 mL/min/1.73 m^2^) during the analysis period. Over the 5-year follow-up period assessed, the cumulative difference in eGFR decline was 8.47 mL/min/1.73 m^2^ in favor of tolvaptan.

Earlier research has provided estimates of tolvaptan benefit over long-term treatment, including a single-center study by Edwards et al^6^ assessing patients with an average of approximately 5 years of follow-up. The study reported here provides a novel contribution by evaluating long-term effects specifically in younger patients, who have been less studied than older populations with ADPKD. The long-term data reported by Edwards et al^6^ was for a comparatively older group (mean age 44 years). A prespecified subgroup analysis of the TEMPO 3:4 clinical trial assessed efficacy in patients aged <35 years but over a shorter follow-up period of only 3 years.^1^ Data pooling allowed us to create a larger analysis population than would have been feasible with individual studies alone and the ability to link longitudinal data for individual participants across sequential studies extended the duration of follow-up. Patient matching and mixed-effects modeling were used to decrease the potential of bias in comparing participants from different studies. These techniques demonstrated utility in estimating the long-term efficacy of tolvaptan in the overall population of patients at increased risk of rapid ADPKD progression^8^; hence, those methods were applied here specifically to a subgroup of younger adult patients.

However, it is essential to acknowledge the limitations of our analysis. The median duration of follow-up was shorter in the control cohort compared with the tolvaptan cohort (median [range] of 1.7 [0.3-5.5] years vs 4.6 [0.0-5.5] years). This disparity primarily arises from the inclusion of patients from OVERTURE, which had a relatively short duration of follow-up. Nonetheless, the control cohort still included 60 patients (29.4%) with long-term follow-up from CRISP and HALT-PKD, and we prioritized matching these patients first (Supplementary Table S1). Furthermore, a sensitivity analysis that excluded patients enrolled in OVERTURE continued to demonstrate a significant eGFR benefit with tolvaptan. Another limitation of the matched analyses is that kidney imaging data and hence Mayo Imaging Classification were not available for all matched patients. Additionally, our extrapolations of eGFR over 35 years assumed a linear model of eGFR decline and retained the same treatment effect observed during the first 5.5 years. This assumption may not accurately reflect real-world progression in the clinic. A linear model of eGFR decline is appropriate for the most rapid progressors (Mayo Imaging Class 1E), whereas patients at lower risk of rapid progression experience a curvilinear trajectory.^18^ The prediction intervals for the estimates of time to kidney failure were broad, with considerable overlap between the 2 cohorts. The extrapolations were provided to visualize the potential benefit for patients in time to kidney failure.^19^ The predicted value should be interpreted with caution.

Although these limitations necessitate a cautious interpretation of the results, it is crucial to emphasize that the pooled database and analysis were designed to maximize duration of follow-up. Therefore, the results presented here represent the best available data on tolvaptan use in young adults with ADPKD who are at heightened risk of rapid progression. Our findings consequently offer valuable insights, which along with discussions of potential treatment risks, can support informed treatment decision-making for young adult patients contemplating the initiation of tolvaptan therapy.
