# Supplementary material for: Tolvaptan and Autosomal Dominant Polycystic Kidney Disease Progression in Individuals Aged 18-35 Years: A Pooled Database Analysis
Source: Kidney Med. 2024 Nov 14;7(1):100935. doi: 10.1016/j.xkme.2024.100935 (PMC11731472; doi:10.1016/j.xkme.2024.100935)
Supplement: Supplementary File (PDF) — Fig S1; Tables S1-S2. [file mmc1.pdf]

Figure S1. Estimates from the mixed model for (**a**) annual rate of eGFR change and (**b**) cumulative eGFR change at years 1, 3, and 5 from "baseline" in the tolvaptan and control cohorts of the sensitivity analysis that excluded participants from OVERTURE

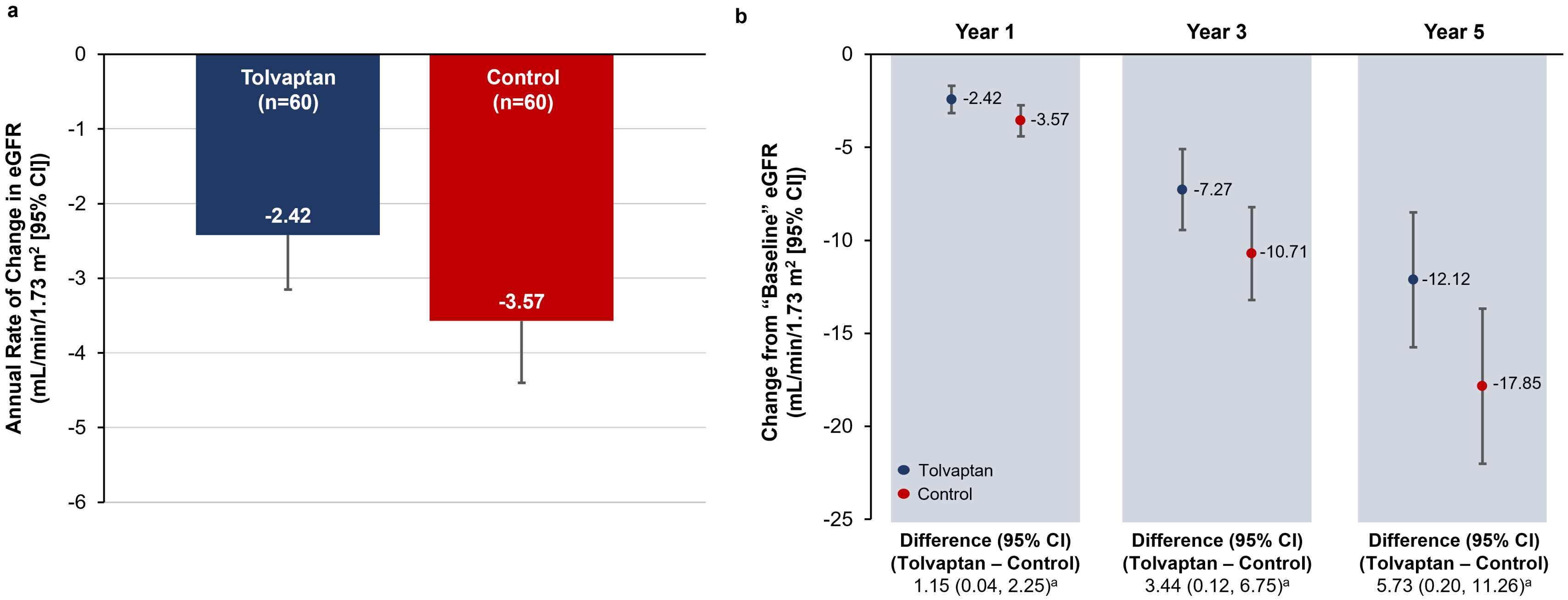

[a] *P*=0.04.

For panel b, change from "baseline" eGFR was calculated based on the theoretical baseline value estimated from the mixed model.

CI, confidence interval; eGFR, estimated glomerular filtration rate.

Table S1. Number of patients matched at each step of the matching procedure

	Tolvaptan[a]		Non–tolvaptan-treated Controls[b]			
	**REPRISE**	**TEMPO 3:4**	**CRISP**	**HALT-PKD Study A**	**HALT-PKD Study B**	**OVERTURE**
**Prematch set:** Aged 18–35 years with a baseline and ≥1 postbaseline eGFR assessment	50	213	108	92	21	615
**Step 1:** Match REPRISE and HALT-PKD Study B, without MIC	7	N/A	N/A	N/A	7	N/A
**Step 2:** Match TEMPO 3:4 to CRISP and HALT-PKD Study A, with MIC	N/A	52	22	30	N/A	N/A
**Step 3:** Match the remaining patients from TEMPO 3:4 to OVERTURE, with MIC	N/A	128	N/A	N/A	N/A	128
**Step 4:** Match the remaining patients from REPRISE to the remaining patients from CRISP, HALT-PKD Study A, and OVERTURE, without MIC	17	N/A	1	0	N/A	16
**Matched analysis set**	24	180	23	30	7	144

[a] Including patients who were randomized to the tolvaptan arm; excluding patients who were enrolled in Japan in TEMPO 3:4. [b] Excluding patients who used tolvaptan, were linked in an early study, were randomized to low blood pressure control in HALT-PKD Study A, or were enrolled in Japan in OVERTURE.

Note: Patients were matched on CKD stage, sex, age (±2 years), eGFR (±5 mL/min/1.73 m^2) and MIC (when available).

CKD, chronic kidney disease; eGFR, estimated glomerular filtration rate; MIC, Mayo Imaging Class; N/A, not applicable.

Table S2. Patient baseline characteristics of the matched set in the sensitivity analysis that excluded participants from OVERTURE

Characteristic	Tolvaptan (n=60)	Non–tolvaptan-treated Controls (n=60)	Standardized Mean Difference[d]
Age in years, mean (SD)	30.3 (4.0)	30.4 (3.9)	-0.04
Range	18.8, 35.0	18.7, 35.0	
Sex, n (%)			
Female	21 (35.0)	21 (35.0)	0.00
Male	39 (65.0)	39 (65.0)	0.00
Race, n (%)			
Asian	0 (0.0)	1 (1.7)	-0.18
Black	2 (3.3)	1 (1.7)	0.11
Hispanic	2 (3.3)	3 (5.0)	-0.08
White	56 (93.3)	50 (83.3)	0.32
Combined[a]	0 (0.0)	5 (8.3)	-0.43
Body mass index in kg/m^2, mean (SD)	26.4 (9.1)	27.7 (6.3)	-0.16
Age at ADPKD diagnosis in years, mean (SD)	22.0 (7.3)	22.3 (7.1)	-0.04
Median (IQR)	23.0 (16.5–28.0)	23.0 (17.9–26.8)	
CKD stage,[b] n (%)			
G1	39 (65.0)	39 (65.0)	0.00
G2	13 (21.7)	13 (21.7)	0.00
G3a	7 (11.7)	7 (11.7)	0.00
G3b	0 (0.0)	0 (0.0)	
G4	1 (1.7)	1 (1.7)	0.00
G5	0 (0.0)	0 (0.0)	
eGFR in mL/min/1.73 m^2, mean (SD)	94.6 (23.7)	94.7 (23.2)	-0.00
Systolic blood pressure in mmHg, mean (SD)	126.0 (13.0)	127.6 (12.7)	-0.12
Diastolic blood pressure in mmHg, mean (SD)	79.7 (8.7)	80.3 (10.1)	-0.06
History of nephrolithiasis, n (%)	12 (20.0)	6 (10.0)	0.28
History of hematuria, n (%)	22 (36.7)	11 (18.3)	0.42
History of urinary tract infection, n (%)	16 (26.7)	7 (11.7)	0.39
TKV in mL, n[c]	52	52	
Mean (SD)	1261.7 (419.8)	1291.1 (472.6)	-0.07
Median (IQR)	1132.8 (942.7–1452.7)	1224.0 (949.3–1541.9)	
Height-adjusted TKV in mL/m, n (%)[c]	52	52	

<400	0 (0.0)	2 (3.8)	-0.28
400 to <600	25 (48.1)	17 (32.7)	0.32
≥600	27 (51.9)	33 (63.5)	-0.24
Mayo Imaging Class, n (%)[c]	52	52	
1A	0 (0.0)	0 (0.0)	
1B	0 (0.0)	0 (0.0)	
1C	16 (30.8)	16 (30.8)	0.00
1D	22 (42.3)	22 (42.3)	0.00
1E	14 (26.9)	14 (26.9)	0.00
Duration of follow-up in years, median (min, max)	5.3 (0.1, 5.5)	5.0 (0.3, 5.5)	[e]

[a] Includes American Indian or Alaska Native, Native Hawaiian or other Pacific Islander, and a race or ethnicity not listed. [b] Stage G1, ≥90 mL/min/1.73 m^2; stage G2, 60 to <90 mL/min/1.73 m^2; stage G3a, 45 to <60 mL/min/1.73 m^2; stage G3b, 30 to <45 mL/min/1.73 m^2; stage G4, 15 to <30 mL/min/1.73 m^2; stage G5, <15 mL/min/1.73 m^2.

[c] The control cohort was restricted to patients whose matched tolvaptan counterparts had nonmissing data. [d] Values >0.2 were considered to be indicative of between-group differences. On the interpretation of standardized mean difference, see: Austin PC. *Stat Med*. 2009;28(25):3083–3107. [e] Standardized mean difference between the treatment cohorts was not calculated, as duration of follow-up is not a baseline characteristic.

ADPKD, autosomal dominant polycystic kidney disease; CKD, chronic kidney disease; eGFR, estimated glomerular filtration rate; IQR, interquartile range; max, maximum; min, minimum; SD, standard deviation; TKV, total kidney volume.
